# Treating traumatic injuries of the diaphragm

**DOI:** 10.4103/0974-2700.62122

**Published:** 2010

**Authors:** Sankalp Dwivedi, Pankaj Banode, Pankaj Gharde, Manisha Bhatt, Sudhakar Ratanlal Johrapurkar

**Affiliations:** Jawaharlal Nehru Medical College, Sawangi, Wardha Maharashtra, India; 1 Datta Meghe Institute of Medical Sciences, Sawangi, Wardha Maharashtra, India

**Keywords:** Blunt diaphragmatic rupture, hemopneumothorax, herniation and strangulation, thoracotomy

## Abstract

Traumatic diaphragmatic injury (DI) is a unique clinical entity that is usually occult and can easily be missed. Their delayed presentation can be due to the delayed rupture of the diaphragm or delayed detection of diaphragmatic rupture, making the accurate diagnosis of DI challenging to the trauma surgeons. An emergency laparotomy and thorough exploration followed by the repair of the defect is the gold standard for the management of these cases. We report a case of blunt DI in an elderly gentleman and present a comprehensive overview for the management of traumatic injuries of the diaphragm.

## INTRODUCTION

The diaphragm is an arched flat muscle that divides the thorax from the abdominal cavity.[1] Although blunt injury of diaphragm is relatively common and is considered as a marker of severe trauma, it can clinically be occult as other violent injuries may mask and disguise its initial clinical presentation.[2,3] An accurate diagnosis requires a high index of suspicion as missed diaphragmatic injury (DI) may result in herniation and strangulation of intra-abdominal viscera into the thoracic cavity.[1] Therefore, the detection, an accurate diagnosis and prompt management of DIs, particularly in severely injured or poly-traumatized patients, becomes a real challenge for the trauma surgeon.[1–5] We report a case of blunt DI in an elderly gentleman managed successfully at our center and present a comprehensive overview for the management of traumatic injuries of the diaphragm.

## CASE REPORT

**Figure 1:**
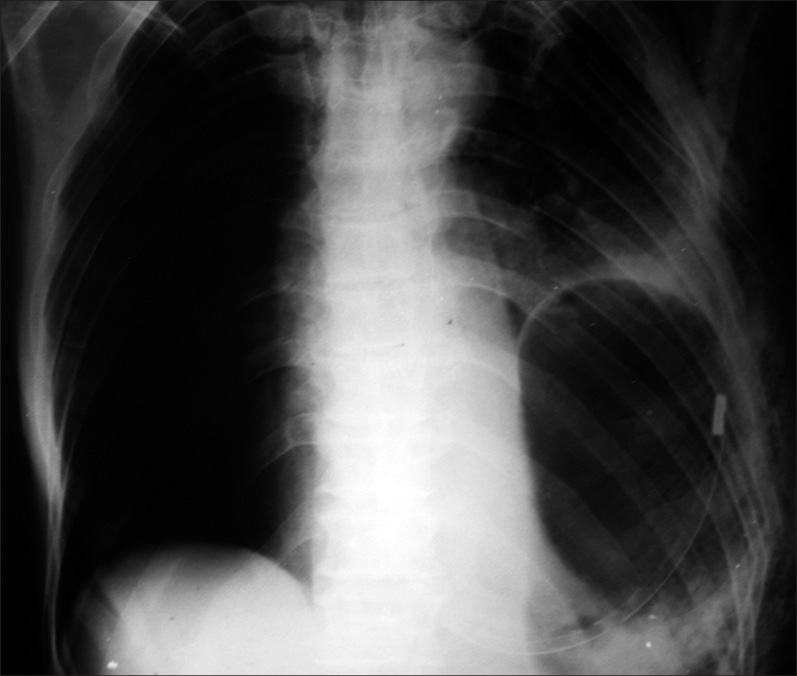
Pre-operative X-ray chest showing left diaphragmatic hernia

**Figure 2:**
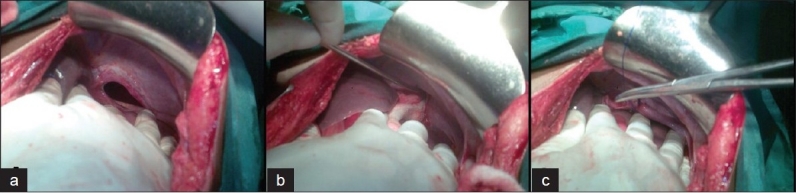
Intra-operative findings suggestive of left diaphragmatic rent with herniation of stomach

**Figure 3:**
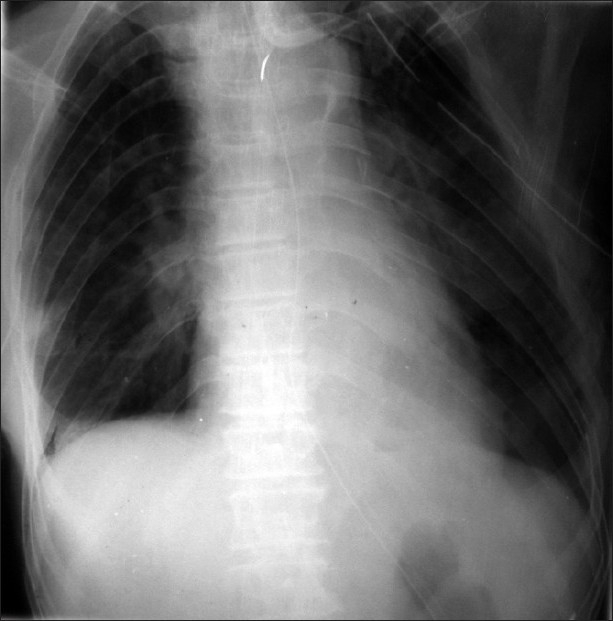
Follow up X-ray

An elderly gentleman was brought to emergency 8 days after blunt thoracoabdominal trauma with diaphragmatic rupture and shock. His chest X-ray showed left hemopneumothorax, multiple rib fractures and elevated left dome of diaphragm with quailed nasogastric tube in the left hemithorax [Figure 1]. The patient was resuscitated and an emergency exploratory laparotomy revealed a 3-cm rent situated posterolaterally on the left dome of the diaphragm with herniation of the stomach into the chest. There were no other associated intra-abdominal injuries. The stomach was healthy and replaced into the abdomen and the diaphragmatic rent was repaired [Figure 2]. The follow-up X-ray of the chest showed full expansion of the left lung and a normally positioned diaphragm [Figure 3]. The patient was doing well on follow-up.

## DISCUSSION

The true incidence of DI is unknown.[5] In 1541, Sennertus first described an autopsy finding of a stomach herniating through a DI. The first anti-mortem diagnosis was made by Bowditch in 1853. Later, the first diagnosis and repair of an acute blunt diaphragmatic rupture was reported in 1900 by Walker. Finally, in 1925, Hedblom conducted a literature-based study consisting of a series of 378 cases, which has been considered as the beginning of the modern surgical era of DI management.[1,4]

### Pathophysiology

Traumatic rupture of the diaphragm may occur due to blunt, penetrating or iatrogenic injuries and only 3-5% of patients require laparotomy.[1] The majority of these injuries occur in men and involve the left hemidiaphragm. Approximately 75% of the diaphragmatic injuries are caused by blunt trauma and 25% by penetrating trauma (ratio ranges from 3:1 to1:8). This is clearly a reflection of the geographic and socioeconomic region served by each hospital.[4] Bilateral DI is uncommon (<2%) (Asensio *et al*., 2004). Blunt trauma occurs mostly due either to road traffic eccedent or falls from a height. This pattern is more common in rural areas.[6] A direct anterior blow to the abdomen leads to a sudden transmission of force through the abdominal viscera that acts as a hydrodynamic fluid wave leading to significantly increase intra-abdominal pressure and disruption.[7] In our case also the mode of injury was the blunt thoracoabdominal trauma due to road traffic accident (RTA) leading to a direct anterior blow to his thorax and abdomen. DI may be associated with herniation of the stomach, small bowel, colon, spleen, liver or omentum.[8] The incidence of right-sided DI is low due to the cushioning effect of the liver.[8,9] It is associated with significant vascular tears in the inferior vena cava or hepatic vein with higher pre-hospital mortality.[8] Gunshot and stab wounds are the major causes of penetrating injuries of the diaphragm. They are potentially more dangerous because they create smaller defects with high probabilities of later obstruction and strangulation.[10] Sometimes during thoracotomy or laparotomy, iatrogenic injuries of the diaphragm may occur, but they are usually insidious and difficult to identify.[1] According to the duration of injury, diaphragmatic rupture can be categorized into one of three groups (early or acute, i.e. immediately or within 14 days post-injury; latent, diagnosed after acute injury but before intestinal obstruction or strangulation; late, where correct diagnosis is established with intestinal obstruction or strangulation). The present case was diagnosed within 8 days and belongs to a group of early or acute one.[5] The missed injuries of the diaphragm may occur either due to delayed rupture or delayed detection. In delayed rupture, the diaphragmatic muscle is devitalized at the time of the initial injury and starts acting as a barrier against herniation. Delayed detection becomes evident only when the intrathoracic pressure becomes negative and herniation occurs some time later. Therefore, during exploratory laparotomy, a meticulous inspection and palpation of the entire diaphragm becomes mandatory in every case of trauma.[4] The diaphragm is rarely injured alone. A triad of severe blunt thoracoabdominal trauma consists of pelvic fractures, blunt diaphragmatic rupture and blunt thoracic aortic rupture.[11–13] According to the literature, a 90% incidence of blunt thoracic injuries such as haemopneumo-thoraces and multiple rib fractures, 40% incidence of associated pelvic fractures, a 25% incidence of both hepatic and splenic injuries and a 5% incidence of associated blunt rupture of the thoracic aorta are associated with DI.[4] The present case showed left hemopneumothorax and multiple rib fractures in association with diaphragmatic rupture and herniated healthy stomach. Other study described a 42% incidence of associated closed head injuries and a 75% incidence of long-bone fractures. Demetriades *et al*. (1988) reported associated intra-abdominal injuries in 122 of 163 patients (75%) sustaining penetrating injuries to the diaphragm in their study.[1]

### Clinical presentation

A high index of clinical suspicion clubbed with imaging studies probably makes an accurate diagnosis of DI. The usual symptoms of DI may include shoulder or epigastric pain, respiratory distress and intrathoracic bowel sounds (Asensio and Petrone, 2004).[1] In cases of delayed presentation with chronic herniation, symptoms of partial or complete intestinal obstruction may be present. Sometimes herniated and distended stomach or colon may easily be mistaken as pneumothorax or hydr-pneumothorax.[5] The clinical signs in the diagnosis of diaphragmatic hernia include diminished expansion of the chest wall, impaired resonance, adventitious sounds, cardiac displacement, circulatory collapse, cyanosis and dyspnea and asymmetry of the hypochondria (Gibson, 1929).[1]

### Diagnosis and investigation

There is no ‘‘gold standard’’ for early diagnosis of traumatic diaphragmatic rupture. Surgical repair is easy before fibrosis develops and the morbidity and mortality associated with any form of delayed diaphragmatic rupture can be avoided. In one study, the insufficient initial evaluation of diaphragmatic hernia with gut in the thoracic cavity led to iatrogenic trauma to the gut and latter contrast computed tomography (CT) showed scattered contrast to the thoracic cavity.[5] Initial chest X-ray is the best diagnostic aid in the evaluation of diaphragmatic rupture. A diaphragmatic hernia should be suspected if X-ray chest shows absence of fundic gas in its normal position, elevation of the hemi diaphragm and absence of a sharp hemi diaphragm or presence of a hemopneimothorax.[4,5] The nasogastric tube placed during the resuscitation may be coiled in the left side of the chest. This classic pattern of elevation and absence of a sharp left hemi diaphragm, hemopneimothorax and coiled nasogastric tube into the thorax on X-ray chest formed the basis of our diagnosis of diaphragmatic hernia without going for further evaluation. Other findings may be curvilinear shadows and air-fluid levels consistent with hollow viscera in the intrathoracic space. These findings are path gnomonic but helpful in diagnosing diaphragmatic rupture in 25-50% cases only.[4] Contrast studies are valuable only in cases of DI with chronic, longstanding herniations of intra-abdominal viscera.[1,14] One study describes the successful use of intraperitoneal technetium to diagnose a suspected rightsided diaphragmatic tear.[4] The diagnostic peritoneal lavage has been used to detect DI in several studies but resulted in falsenegative rates as high as 25-35% when studied retrospectively. This lack of sensitivity may be because the diaphragmatic rupture is the only abdominal injury itself and the negative intrathoracic pressure has a tendency to pull intra-abdominal fluid and blood into the thorax.[4] The real time ultrasonography (US) may occasionally reveal a diaphragmatic rupture in expert hands. Focused Abdominal Sonography for Trauma (FAST) is supposed to be the more accurate technique for trauma settings, but no definitive reports have emerged describing its usefulness in the diagnosis of DI.[1] CT scan is the second choice of imaging technique after X-ray chest that may demonstrate the herniation of the abdominal viscera in the thorax but may not be able to directly image the diaphragmatic lacerations.[5] When chest radiography is indeterminate, spiral CT, preferably with multiple detector rows, is the most appropriate second-line study.[15] Currently, reconstructed images are routinely obtained in patients with chest radiographic findings suspicious of DI (Mirvis and Shanmuganagthan, 2007). The use of multi-detector computed tomography with coronal and sagittal multiplanar reformation has improved the accuracy of CT here.[1] The findings on axial CT Scan e.g. “dependent viscera” sign or “Hump on Band” are realy conclusive for DI.[3] When CT results remain equivocal, T1-weighted sagittal and coronal magnetic resonance imaging has proven very useful for both right- and left-sided injury.[15]

### Thoracoscopy and laparoscopy

Laparoscopy and thoracoscopy are now the diagnostic and therapeutic choices of trauma surgeons. Laparoscopy allows assessment and repair of the diaphragm.[1,14] If open surgery is not required, suspected patients should undergo either diagnostic laparoscopy or video-assisted thoracoscopy.[5] Thoracoscopy is probably more useful in obvious thoracic injuries and in right-sided penetrating thoracoabdominal injuries. In a prospective study, penetrating injuries to the left lower chest not requiring laparotomy, routine diagnostic laparoscopy identified occult diaphragmatic injuries in 24% of the patients.[16] Another study conducted in 1993 concluded that the diagnostic accuracy of laparoscopy was excellent for haemoperitoneum, solid-organ injuries and diaphragmatic lacerations in haemodynamically stable patients. Later, Murray *et al*. (1997) found that 26% of the patients undergoing laparoscopy had occult diaphragmatic injuries. Laparoscopy proved itself a vital tool for detecting these injuries among patients who had no other indications for laparotomy.[17] For haemodynamically stable patients, diagnostic laparoscopy is utilized for patients sustaining left thoracoabdominal penetrating trauma. Thoracoscopy is a very sensitive and specific diagnostic tool in DI, with a very high accuracy of between 98 and 100%. Koehler and Smith suggested that combined thoracoscopy and laparoscopy may offer both therapeutic as well as diagnostic benefit in selected stable patients with penetrating injuries to the upper abdomen and lower chest. The main disadvantages of thoracoscopy are the amount of time it takes to place the patient in a thoracotomy position, not allowing repair of the diaphragm, and that it always requires a chest tube insertion even if negative (Asensio and Petrone, 2004). The patients with penetrating injuries to the left lower chest and having no indication for a laparotomy should undergo videoscopic evaluation of the left hemidiaphragm.[16] Video-thoracoscopy (VATS) can be beneficial to hemodynamically stable patients but requires evaluation.[5]

### Treatment

The evaluation of the diaphragm by laparotomy remains the gold standard for diagnosis. Repair of the diaphragmatic ruptures can be performed by the classical open method or with minimally invasive methods. Open exploration can be carried out through the abdominal or the thoracic route.[5,14] In case of acute injuries, like in polytrauma, the basic resuscitative measures of Advanced Trauma Life Support of the Trauma Care Manual (1997) should be applied first followed by the insertion of a nasogastric tube and a thoracostomy tube if required. The basic principles of trauma surgery, like control of hemorrhage and shock, should be closely followed-up, as we did in our case also. At laparotomy, careful inspection of the diaphragm may require transaction of the falciform ligament and gentle downward traction of the liver for the right hemi diaphragm and gentle downward retraction of the spleen and greater curvature of the stomach for the left hemi diaphragm. Finally, diaphragmatic lacerations can be repaired with non-absorbable sutures. In cases of diaphragmatic disruption due to massive trauma, prosthetic non-absorbable mesh material is used to reconstruct the diaphragm. In the majority of cases, chest tube drainage is required. Patients of initially sustained small and undetected DI usually show an increase in visceral herniation and latter obstruction, and even strangulation. The operative repair of a rupture is simple if performed immediately. We successfully repaired the diaphragmatic rent with intermittent polypropylene suture. Delayed repair becomes difficult mainly because of adhesions and atrophy of the diaphragm and subject to higher chances of dehiscence. The non-absorbable prosthetic meshes are required to repair such chronic injury. An extensive review of the literature revealed that 74% of the total DI was repaired via laparotomy, while 18% were via thoracotomy and 8% had thoracoabdominal approaches.[1,5]

### Complications

The most serious complication of DI is perforation of viscera into the thoracic cavity, leading to infections like pneumonia, empyemas and sub-phrenic or intra-abdominal abscesses. To avoid this, the vigorous irrigation of the thoracic and abdominal cavity with adequate drainage is recommended.[4,5] The morbidity includes complications like suture-line dehiscence, hemi diaphragmatic paralysis secondary to iatrogenic phrenic nerve injuries, respiratory insufficiency, empyemas and subphrenic abscess. The underlying trauma and associated injuries may be the cause of late morbidity.[1] Pulmonary embolism is now rare as a result of the aggressive use of prophylaxis.[4]

### Mortality

The overall mortality rate reported in the literature ranges from 4.3 to 37% in a series of penetrating and blunt injuries, respectively. Irreversible shock and head injury are most likely causes of intra-operative or early post-operative deaths whereas sepsis and multisystem organ failure predominate as late causes of death.[1,4]

## CONCLUSION

Traumatic injuries of the diaphragm are often clinically occult and can be masked and disguised by other violent injuries associated with polytrauma. The best approach is the high index of suspicion in such cases.[4] Chest X-ray is the initial screening option followed by spiral CT (preferably with multidetector rows) to evaluate the diaphragm. Optimal treatment of DI consists of early repair on laparotomy with careful evaluation of other associated violent injuries;[1,5] however, with an increase in experience and expertise, laparoscopy and thoracoscopy, especially VATS, are finding their places in both diagnosis and definitive management of thoracic trauma with occult diaphragmatic injuries.[16]
